# Fiberscopic Imaging of the Pediatric Nasopharynx

**DOI:** 10.1155/DTE.1.153

**Published:** 1995

**Authors:** Deyun Wang, Peter Clement

**Affiliations:** Department of Otorhinolaryngology University Hospital Free University of Brussels Laarbeeklaan 101 Brussels 1090 Belgium

## Abstract

This study describes the endoscopic findings about the size of the adenoid tissue and the condition of the nasopharyngeal orifice of the eustachian tube. Results confirmed that only fiberscopic examination allows a thorough inspection of the nasopharyngeal anatomy to make a correct diagnosis and design therapeutic planning. When the presence of adenoid hypertrophy resulting in nasal obstruction, snoring, and/or otitis media was confirmed endoscopically, adenoidectomy proved to be highly efficacious in relieving these symptoms.


*Diagnostic and Therapeutic Endoscopy,* 1995, Vol. 1, pp. 153–157
Reprints available directly from the publisher
Photocopying permitted by license only

© 1995 Harwood Academic Publishers GmbH
Printed in Malaysia

### Fiberscopic Imaging of the Pediatric Nasopharynx

DEYUN WANG and PETER CLEMENT

*Department of Otorhinolaryngology, University Hospital,*
*Free University of Brussels, Laarbeeklaan 101, 1090 Brussels, Belgium*

*(Received March 7, 1994; in final form, June 29, 1994)*

This study describes the endoscopic findings about the size of the adenoid tissue and the condition of the nasopharyngeal orifice of the eustachian tube. Results confirmed that only fiberscopic examination allows a thorough inspection of the nasopharyngeal anatomy to make a correct diagnosis and design therapeutic planning. When the presence of adenoid hypertrophy resulting in nasal obstruction, snoring, and/or otitis media was confirmed endoscopically, adenoidectomy proved to be highly efficacious in relieving these symptoms.

**KEY WORDS:** pediatric fiberscopy, nasopharynx, adenoidectomy, eustachian tubal orifice

### INTRODUCTION

The flexible fiberscope has made accurate evaluation of the condition of the pediatric nasopharynx possible. It is the only means of examination that allows direct visualization of the nasopharynx and enables the definite diagnosis of adenoid hypertrophy. The findings of fiberoptic examination of the nasopharynx correlate well with the results of tympanometry and radiography and lead to a considerable improvement in the accuracy of the determination of indication for adenoidectomy (1).

This study discusses fiberscopic findings to assess the condition of the nasopharyngeal cavity in pediatric patients. It mainly focuses on the size and shape of the adenoid tissue and the condition of the eustachian tubal orifice in relation to the complaints of chronic nasal obstruction, snoring, and otitis media accompanied by effusion.

### MATERIALS AND METHOD

### Instruments

A flexible fiberoptic bronchoscope (BF type 3c10 or 3c20, Olympus, Tokyo, Japan) with a TVR 4500 color video camera (Switzerland) was used to examine the nasal cavity and nasopharynx to record all examinations and to reassess them later.

### Patients

From December 1989 to December 1991, 371 children (227 boys and 148 girls) age 26 days to 14 years (mean age: 5.34 years) were included in this study. All patients were referred from the pediatric ENT outpatient clinic. Two hundred eighty-four children had complaints of nasal obstruction (mouth breathing, with or without snoring). The control group consisted of 91 children without complaints of nasal obstruction. In this group, fiberscopic examination of the nasal cavity and nasopharynx, and/or larynx was performed because of clinical necessity. Children with an acute infection of the respiratory tract (associated with fever) were excluded from this study. All parents gave their consent and were invited to attend the examination of their children. This study was approved by the local ethics committee.

### Anesthesia

A cotton strip soaked with a mixture of 1% Novesine (oxybuprocaine HCL) and 0.05% otrivine (xylometazoline) was applied for local anesthesia and vasoconstriction. Vasoconstriction is especially necessary in children because of the narrowness of the nasal passage and the frequent presence of swelling of the nasal mucous membrane. Most children younger than 1 year and older than 4 years can be examined in this way easily.

Address for correspondence: Deyun Wang, M.D., Department of Otorhinolaryngology, University Hospital, Free University Brussels (AZ-VUB), Laarbeeklaan 101, 1090 Brussels, Belgium.

A combination of Nembutal (Pentobarbitone) suppository and Thalamonal (Droperidol 2.5mg + Fentanyl 50 μg/ml) intramuscular injectioned was scheduled for use in children aged from 1 to 4 years. The authors, however, always first tried to use local anesthesia only.

### Measurements

According to the distance from the vomer to the adenoid tissue, the relative size of the adenoid tissue was judged by fiberscopy, which led to classification into three categories: (1) small, (2) moderate, and (3) large (1). The methods for measuring the condition of the eustachian tubal orifice as well as the evaluations of the tympanogram, the nasal obstruction, and snoring have been described by the authors previously (1–3).

### RESULTS

Endoscopies were performed without complication and completed in 370 of 371 patients. In one infant, 42 days old, it was impossible to introduce the endoscope because of extreme swelling of the nasal mucosa. Figure 1 shows the relative sizes of the adenoid tissue according to our classifications, *i.e*, small, moderate, and large.

There was a higher incidence of large adenoids in the group with continuous nasal obstruction complaints (59.3%) and a distinctly lower incidence in the groups with periodic (22.2%) or no (8%) nasal obstruction complaints (2). A high correlation also was observed between size of the adenoid and snoring ($p < 0.001$) (3) A large adenoid occurred more frequently in the group of patients who snored continuous (65.1%) than in the group of patients who experienced periodic snoring (41.5%) and absence of snoring (23.2%).

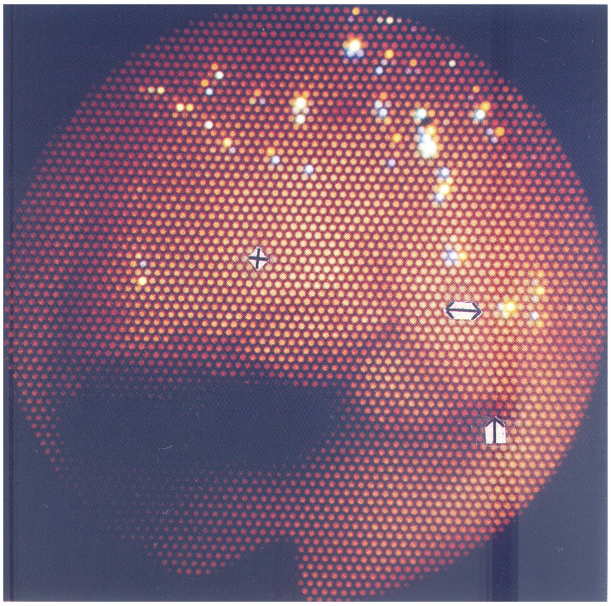

**Figure 1B** Moderate adenoid.

In Figure 2, different nasopharyngeal pathologies are shown in patients who were referred to our clinic with nasal and/or ear complaints. However, a causative factor of adenoid hypertrophy was suspected. Endonasal

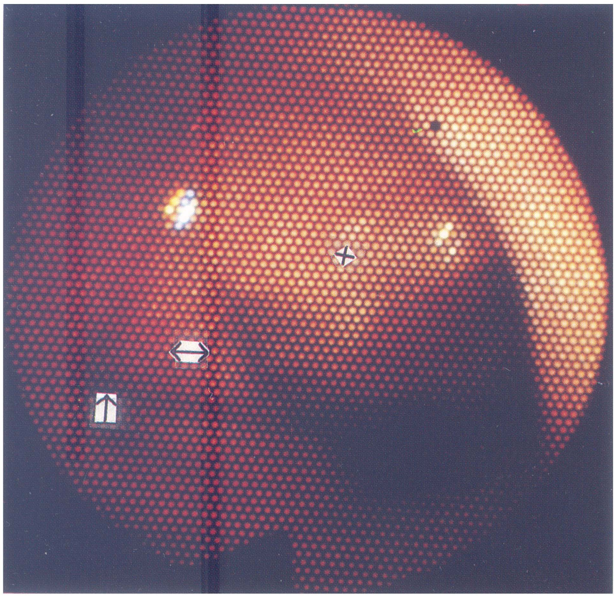

**Figure 1** Size of the adenoid tissue (+: adenoid, ↑: nasopharyngeal orifice of the eustachian tube, ↔: torus tubarius). A, small adenoid.

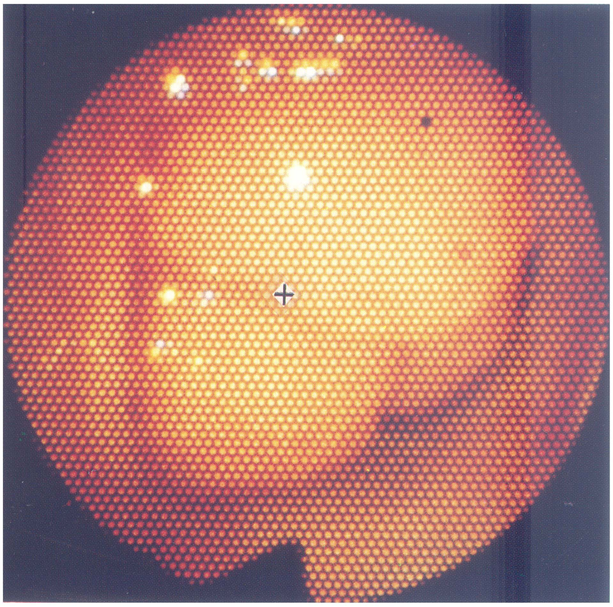

**Figure 1C** Large adenoid (adenoid hypertrophy).

fiberscopy of the nasal cavity and nasopharynx was, therefore, required. A 4-year-old girl had complaints of hearing difficulty and a history of continuous mouth breathing and snoring (Fig. 2A). Tympanometry showed a flat curve without minimal impedance (type B) on both sides. Fiberscopic examination performed under local anesthesia with premedication showed enlarged adenoid tissue that completely blocked the choana and the nasopharyngeal orifices of the eustachian tube. Within 1 week after adenoidectomy, snoring had disappeared and normal nose breathing was observed.

Medical advice was sought for a 1-year-old boy because of bilateral otitis media with effusion (Fig. 2B). There was a history of periodic mouth breathing and snoring. The tympanogram showed a flat curve for both ears. Under local anesthesia with premedication, fiberscopy showed a small adenoid tissue. A pus stream from the nasal cavity passed over the patent eustachian tubal orifice. Further examination was recommended to confirm the existence of sinusitis.

A 6-year-old girl presented with continuous mouth breathing, snoring, and hearing loss, although adenoidectomy had been performed 2 1/2 years before (Fig. 2C). An adenoidectomy again was advised by the general physician. Tympanometry suggested the existence of otitis media with effusion in both ears. Fiberscopic examination of the nasal cavity and nasopharynx was performed under local anesthesia only. Minimal adenoid tissue was encountered; however, endoscopy did reveal a swollen torus tubarius that nearly blocked the eustachian tubal orifice and partially blocked the choana.

A 4-year-old boy was referred with continuous mouth breathing, snoring, and hearing loss (Fig. 2D and E). The patient had undergone adenoidectomy on two previous occasions, as well as tonsillectomy and multiple myringotomies with tympanostomy tube insertions (three times)

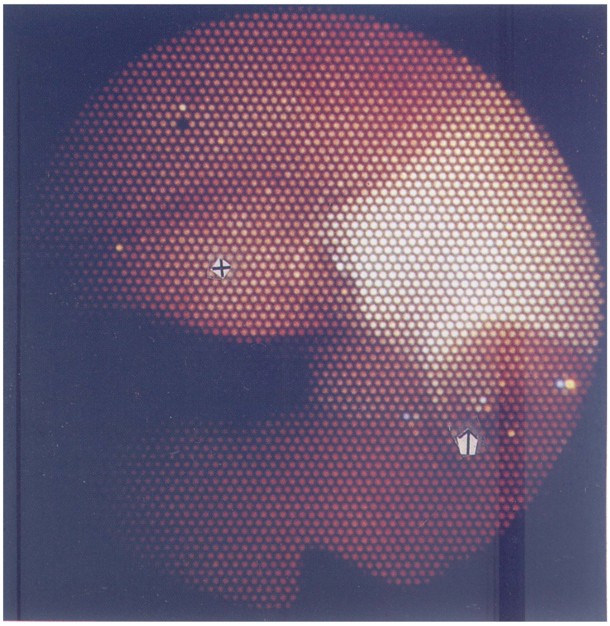

**Figure 2B** With a small adenoid tissue, the eustachian tubal orifice is anatomically open. There are some purulent secretions passing over the tubal orifice.

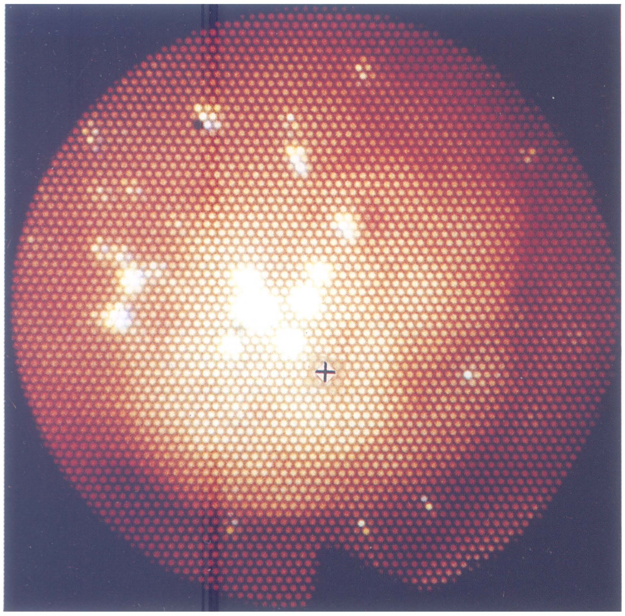

**Figure 2** Different conditions of the nasopharynx (+: adenoid; ↑: nasopharyngeal orifice of the eustachian tube, ↔: torus tubarius). A, large adenoid tissue that has completely blocked the choana as well as the nasopharyngeal orifice of the eustachian tube.

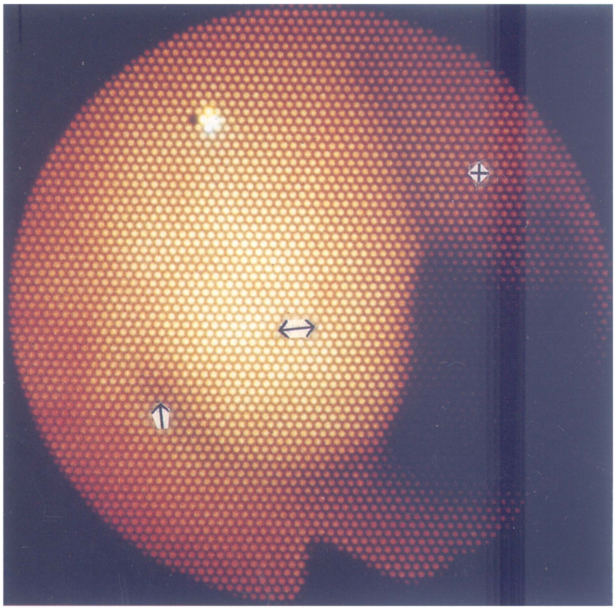

**Figure 2C** Small adenoid tissue but very swollen torus tubarius that blocked the eustachian tubal orifice almost completely as well as partially blocked the choana.

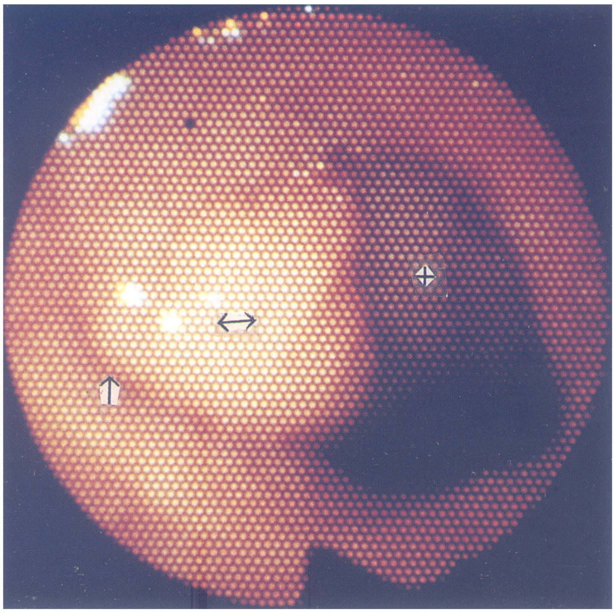

**Figure 2D** Small adenoid but very swollen torus tubarius that blocked the eustachian tubal orifice almost completely.

without benefit. An adenoid hypertrophy again was suspected by one physician. Tympanometry suggested that there was bilateral otitis media with effusions. Fiberscopic examination of the nasal cavity and nasopharynx then was performed under local anesthesia. It revealed a small adenoid tissue but with a markedly swollen torus tubarius that partially blocked the eustachian tubal orifice. On the left side (2-E), mucosal scar tissue was found between the vomer and the torus tubarius. There were abundant purulent secretions in both nasal cavities. These findings indicated that adenoidectomy certainly should be avoided in this patient. The elimination of nasal (probably sinus) infection had to be considered first. This eventually could be followed by appropriate choanal corrective surgery on the left side.

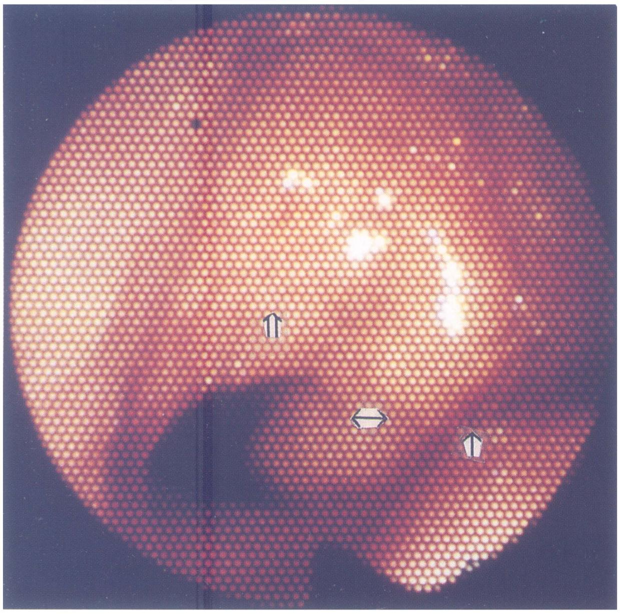

**Figure 2E** Same patient as D. A formation of mucosal scar tissue was found (⇑) at the left side between the Vomer and torus tubarius.

### DISCUSSION

Examination of the ears, nose, nasopharynx, hypopharynx, and larynx has been dramatically altered by the advent of flexible fiberscopic devices (4). The major advantage of the fiberscopic examination is that it provides direct visualization of normal and pathologic conditions. In pediatric patients, of course, the major problem is patient cooperation. In our study, topical anesthesia alone could be used in all children younger than 1 year and older than 6 years and in most of the 4- to 6-year-old children. In most children between 1 and 3 years, a combination of premedication and local anesthesia is needed (1). A careful explanation to the child and a skilled endoscopist, however, are the most important guarantors of a successful pediatric endoscopy.

It is well known that adenoidectomy is one of the most commonly performed surgical treatments in children. Adenoid hypertrophy often has been considered to relate to many disorders of the upper respiratory tract. The authors have reported that the size of the adenoid tissue correlates very well with the complaints of nasal obstruction (1,2), snoring (3), and the type of tympanogram (1). When an adenoid hypertrophy was evidenced by endoscopic examination, nasal obstruction was improved in 77.2% of patients, and snoring was ended in 89% after adenoidectomy. On the other hand, postoperative training for nasal breathing by the child's parents is very important, because hypertrophy of the adenoid can induce habitual mouth breathing.

Adenoid hypertrophy is one of the common etiologic factors in pediatric otitis media. The mechanism may involve direct closure of the nasopharyngeal orifice of the eustachian tube, resulting in middle ear transudate. The authors reported previously that there is a better correlation between the condition of the eustachian tubal orifice found during fiberscopy and the tympanogram of the same side ($P < 0.0001$) than between the size of the adenoid and the tympanogram ($P = 0.009$) (1). It proves the importance of direct visualization by fiberscopy in the diagnosis of otitis media in some children. On the other hand, otitis media has many causes. Recently, many studies demonstrated that the adherence of bacteria, especially the nontypable *Haemophilus influenzae* (NTHI) has become the predominant cause of both acute suppurative otitis media and chronic otitis media with effusion (5–7). As shown in some of our cases, an antiinfectious treatment should be planned first rather than initially attempting surgery.

In summary, the authors conclude that fiberscopy is the only examination that allows direct visualization of the nasal cavity and nasopharynx and that it is especially useful for the visualization of the local condition of the nasopharynx. It is the only examination that allows differentiation of adenoid hypertrophy, infection, and scar formation. When the presence of adenoid hypertrophy resulting in nasal obstruction, snoring, and/or otitis media is confirmed, adenoidectomy proves to be highly efficacious in relieving these symptoms.

### ACKNOWLEDGEMENTS

The authors thank Peter Lottefier for his contribution to this article.

### REFERENCES

1. Wang D, Clement P, Kaufman L, et al. Fiberoptic examination of the nasal cavity and nasopharynx in children. Int J Pediatr Otolaryngol, 1992;24:35–44
2. Wang D, Clement P, Kaufman L, et al. Chronic nasal obstruction in children. A fiberscopic study. Rhinology, 1994 (in press)
3. Wang D, Clement P, Kaufman L, et al. Fiberoptic evaluation of the nasal and nasopharyngeal anatomy in children with snoring. J Otolaryngol, 1994;23:57–60
4. Selkin SG. Flexible fiberoptics and pediatric otolaryngology: a simple technique for examination and photodocumentation. Int J Pediatr Otolaryngol, 1983;5:325–333
5. Murphy TF, Bernstein JM, Dryja DM, et al. Outer membrane protein and lipooligosaccharide analysis of paired nasopharyngeal and middle ear isolates in otitis media due to nontypable *Haemophilus influenzae*: pathogenetic and epidemiological observation. J Infect Dis, 1987;156:723–731
6. Bernstein IM, Dryja DM, Loos BG, et al. Restriction fragment mapping of nontypable *Haemophilus influenzae*: a new tool to study this middle ear pathogen. Otolaryngol Head Neck Surg, 1989;100:200–206
7. Faden H, Bernstein J, Brodsky L, et al. Otitis media in children, I: The systemic response to nontypable *Haemophilus influenzae*. J Infect Dis, 1989;160:999–1004

